# Diabetic retinopathy screening and treatment through the Brazilian National Health Insurance

**DOI:** 10.1038/s41598-022-18054-6

**Published:** 2022-08-17

**Authors:** Arthur Gustavo Fernandes, Aline Nunes Ferraz, Rodrigo Brant, Fernando Korn Malerbi

**Affiliations:** 1 Department of Ophthalmology and Visual Sciences, Paulista Medical School, Federal University of Sao Paulo, Rua Botucatu, 816, São Paulo, SP 04023-062 Brazil; 2 Department of Anthropology and Archaeology, University of Calgary, Calgary, AB Canada

**Keywords:** Health care, Eye diseases, Diabetes complications

## Abstract

The current study aimed to investigate diabetic retinopathy (DR) screening and treatment coverages among diabetic patients evaluated through the Brazilian National Health Insurance from 2014 to 2019. The Brazilian Public Health System Information Database was used as the primary data source. DR screening coverage was calculated as the rate of procedures of clinical dilated fundus exam and color fundus photograph over the number of diabetic patients. DR treatment coverage was calculated as the rate of procedures of intravitreal injection, photocoagulation, and panretinal photocoagulation over the number of diabetic patients presumably in need of DR treatment. The overall screening coverage increased from 12.1% in 2014 to 21.2% in 2019 (*p* < 0.001) with substantial regional discrepancies so that North region was the only one with no changes along the period. The overall treatment coverage increased from 27.7% in 2014 to 44.1% in 2019, with Southeast and Midwest absorbing the demand for service from the North, Northeast and South. Despite an improvement along the past years, both screening and treatment coverages for DR in diabetes patients are ineffective in Brazil. Public health policies should address resources disparities throughout the country aiming to offer same healthcare conditions to patients regardless their geographic location.

## Introduction

Diabetes mellitus (DM) is one of the most common chronic diseases worldwide with an estimated prevalence of 9.3% of the adult population in 2019^1^. DM is a chronic metabolic disorder characterized by elevated levels of plasma glucose^2^. The disease can be classified into two main forms of diabetes: type 1 diabetes, when pancreatic beta cells are destroyed and consequently there is no insulin production; and type 2 diabetes, when the organism develops insulin resistance leading to hyperglycemia^2^. Chronic hyperglycemia due to DM may lead to different micro (nephropathy, neuropathy, and retinopathy) and macrovascular (peripheral artery disease, ischemic heart disease, and stroke) complications. Interventions focused on glucose levels control as well as lifestyle changing are highly recommended in order to reduce those manifestations incidence^3^.

Diabetes Retinopathy (DR) is the most common and specific DM complication, and one of the leading causes of preventable blindness in the adult population^4, 5^. DR can be classified as non-proliferative diabetic retinopathy (NPDR) as an early stage, which can be mild, moderate, or severe; as proliferative DR (PDR) as the severe stage; and as clinically significant macular oedema (CSME)^6^. A recent meta-analysis estimates the prevalence of DR on 22.27% (95% Confidence Interval [CI]: 19.73%–25.03%) globally within the DM population. Moreover, it shows a prevalence of 6.17% (95% CI, 5.43%–6.98%) for severe NPDR and PDR, and 4.07% (95% CI, 3.42%–4.82%) for CSME, those considered vision-threatening conditions^7^. Vision loss from DR could be prevented with a broad-based systems-level approach: first, by increasing patient’s awareness with specific health education programs; second, by community-level and/or national screening programs for all diabetic patients; and third, by timely referral and treatment for severe stages of DR^6^.

The ideal screening should include a complete ocular examination with best-corrected visual acuity after refraction and a retinal imaging with wide-field retinal photography and optical coherence tomography under pupil dilatation. This approach, however, is not always feasible even in high-resource settings. Recent guidelines recommend a screening including presenting visual acuity and a retinal examination adequate for DR classification, either a clinical dilated fundus exam or a color fundus photographs, depending on the resource settings^6^. In terms of treatment, previous randomized clinical trials have shown that up to 98% of blindness due to RD could be prevented by timely treatment with laser photocoagulation therapy, vitrectomy surgery, or intraocular injections of anti-vascular endothelial growth factor (VEGF) inhibitors^6, 8–10^.

Brazil is a country that offers universal, free of charge health insurance financed by the central government (Sistema Único de Saúde—SUS), providing ocular medical attention to the entire Brazilian population including RD screening and treatment. All the national data related to SUS is centered in the Brazilian Public Health System Information Database (DATASUS)^11^. Previous studies have shown that 3/4 of Brazilian citizens use SUS as their primary health provider while 1/4 rely on private hospitals or private insurance system facilities^12–15^. Recently, a study on retinal exams through SUS has shown that about 1/4 of those procedures are performed as diabetic related complications screening^16^.

The purpose of this study is to investigate the diabetic retinopathy screening and treatment coverages among diabetic patients evaluated through the Brazilian National Health Insurance (SUS) throughout the country from 2014 to 2019 as well as to evaluate each region capacity to attend its treatment demand considering intravitreal injection, photocoagulation, and panretinal photocoagulation.

## Methods

Brazilian Public Health System Information Database (DATASUS) was used as the primary data source for the current study. DATASUS represents the primary effort of Brazilian Federal Government to collect data from the national health system and includes information from all public health hospitals throughout the country.

Screening procedures of clinical dilated fundus exam and color fundus photographs were retrieved for the current analysis. The numbers of procedure were adjusted accordantly to those performed as diabetic related complications screening. Data on the number of diabetic patients in the country were obtained from the Brazilian Risk Factor Surveillance System for Chronic Diseases (VIGITEL) Program prevalence estimates^17^. The coverage of screening test among diabetic patients was calculated considering the numerator as the number of diabetic retinopathy screening exams (1/4 of total)^12–15^ and the denominator the number of diabetes patients who use SUS (3/4 of total)^16^, through the formula below:

$$Screening\, \, Coverage \, = \frac{Total\, \, Screening\, \, Exams\, \, through\, \, SUS\, \, in\, \, the\, \, region \, * \, 0.25}{{Total\, \, Number\, \, of\, \, Diabetic\, \, patients\, \, in\, \, the\, \, region*0.75}}$$

The total number of treatment procedures including injection, photocoagulation, and panretinal photocoagulation were analyzed according to year, region where the procedure was carried out, and region of the patient’s residence. The treatment coverage among diabetic patients was calculated considering the numerator as the number of diabetic retinopathy treatment procedures and the denominator the number of diabetes patients who use SUS (3/4 of total) presumably in need of diabetic retinopathy treatment (10%), through the formula below:

$$Treatment\, \, Coverage \, = \frac{Total\, \, Treatment\, \, procedures\, \, through\, \, SUS\, \, in\, \, the\, \, region}{{Total\, \, Number\, \, of\, \, Diabetic\, \, patients\, \, in\, \, the\, \, region*0.75*0.10}}$$

Spatial treatment coverage was defined as the capacity to attend the treatment demand for each region and it was calculated as the rate of performed procedures in the region over the number of residents from that region submitted to the procedure anywhere through the formula below:

$$Spatial\, \, treatment\, \, coverage \, = \frac{Total\, \, of\, \, procedures\, \, performed\, \, in\, \, the\, \, region}{{Total\, \, of\, \, the\, \, region^{\prime}s\, \, residents\, \, who\, \, received\, \, treatment\, \, anywhere}}$$

The study was conducted exclusively with publicly available data from DATASUS (https://tabnet.datasus.gov.br) without any type of identification of subjects and it was carried out in accordance with the tenets of the Declaration of Helsinki.

Statistical analyses were performed using Stata/SE Statistical Software, Release 14.0, 2015 (Stata Corp, College Station, Texas, USA). Frequency tables were used for descriptive analysis. Trends along the years were evaluated through univariate generalized linear models. Maps were created using the web application go-cart.io^18^. *p* values ≤ 0.05 were considered statistically significant.

## Results

### Screening

**Figure 1:**
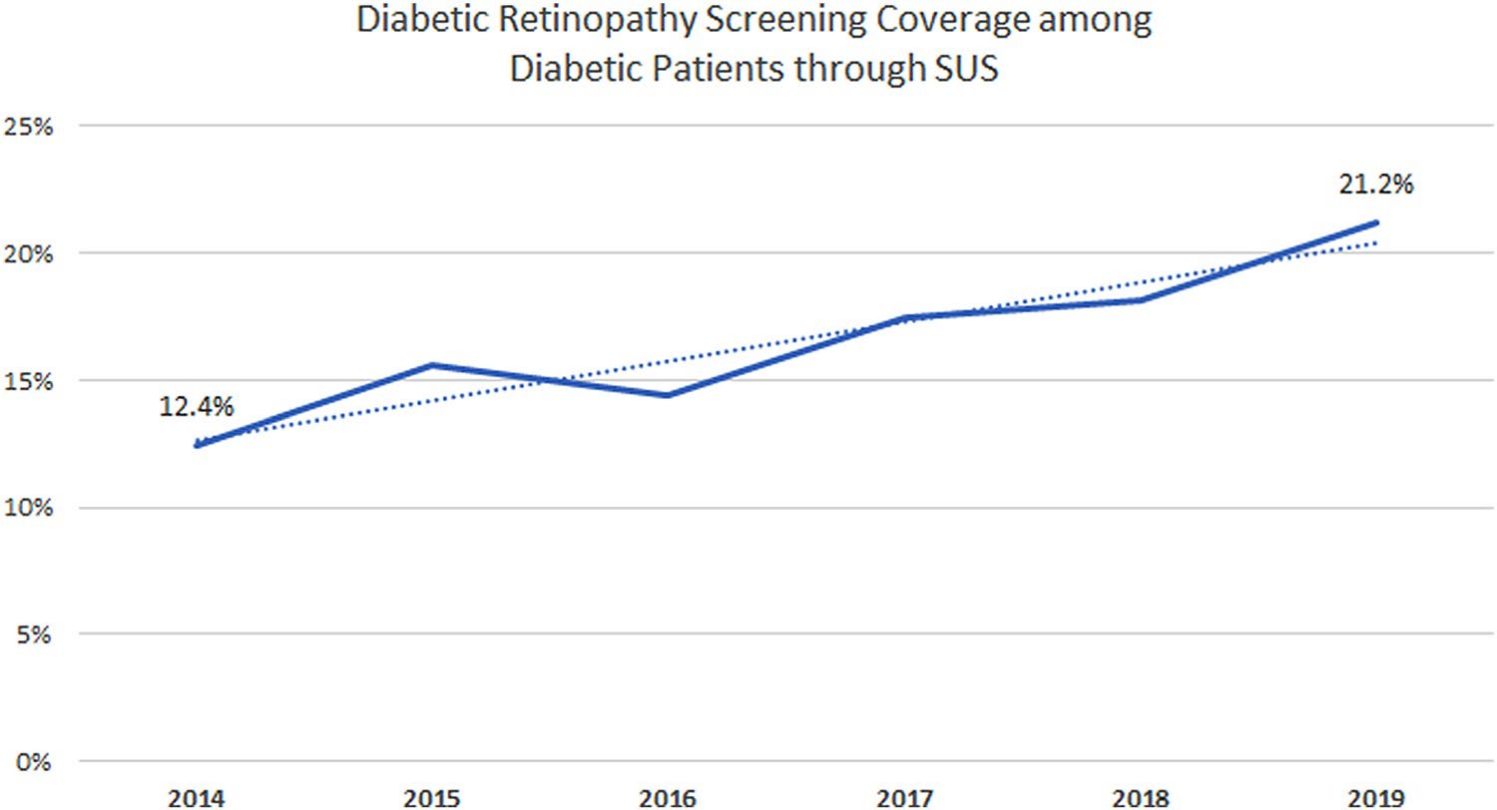
Diabetic retinopathy screening coverage among diabetic patients through SUS from 2014 to 2019.

Along the 6 years period from 2014 to 2019, a total of 21,325,380 screening retinal tests were performed through the National Health Insurance (SUS). The total estimated to be associated with diabetic retinopathy screening was 5,331,345 procedures. Figure 1 shows the trends in screening exams coverage of diabetic patients evaluated by SUS along the years.

Trend analysis shows a statistically significant increase on the coverage along the years (*p* = 0.004). When comparing 2019 to 2014, a change of 71.0% has occurred. The coverages, however, vary substantially according to the country region. Table 1 shows the number of procedures and coverage along the years in the different regions.

**Table 1:** Number of procedures and coverage of Diabetic Retinopathy Screening among Diabetic Patients through SUS from 2014 to 2019 according to the region.

	North	Northeast	Southeast	South	Midwest	All
2014	30,364 7.8%	194,713 14.9%	300,924 12.0%	105,853 13.1%	38,843 11.2%	670,697 12.4%
2015	32,267 9.1%	212,865 16.7%	353,020 15.8%	150,638 20.2%	40,257 11.2%	789,047 15.6%
2016	28,261 7.8%	241,479 15.5%	390,100 13.4%	165,779 19.9%	63,640 12.8%	879,258 14.4%
2017	30,053 7.9%	237,783 18.3%	420,846 17.3%	181,852 26.0%	50,557 13.6%	921,089 17.5%
2018	30,716 5.7%	240,007 17.4%	441,862 18.8%	202,307 28.6%	56,373 15.9%	971,265 18.1%
2019	32,661 8.8%	277,310 20.4%	488,055 20.9%	245,872 33.3%	56,093 15.3%	1,099,991 21.2%

First row: Number of diabetic retinopathy screening procedures.

Second row: Diabetic Retinopathy Screening Coverage.

**Figure 2:**
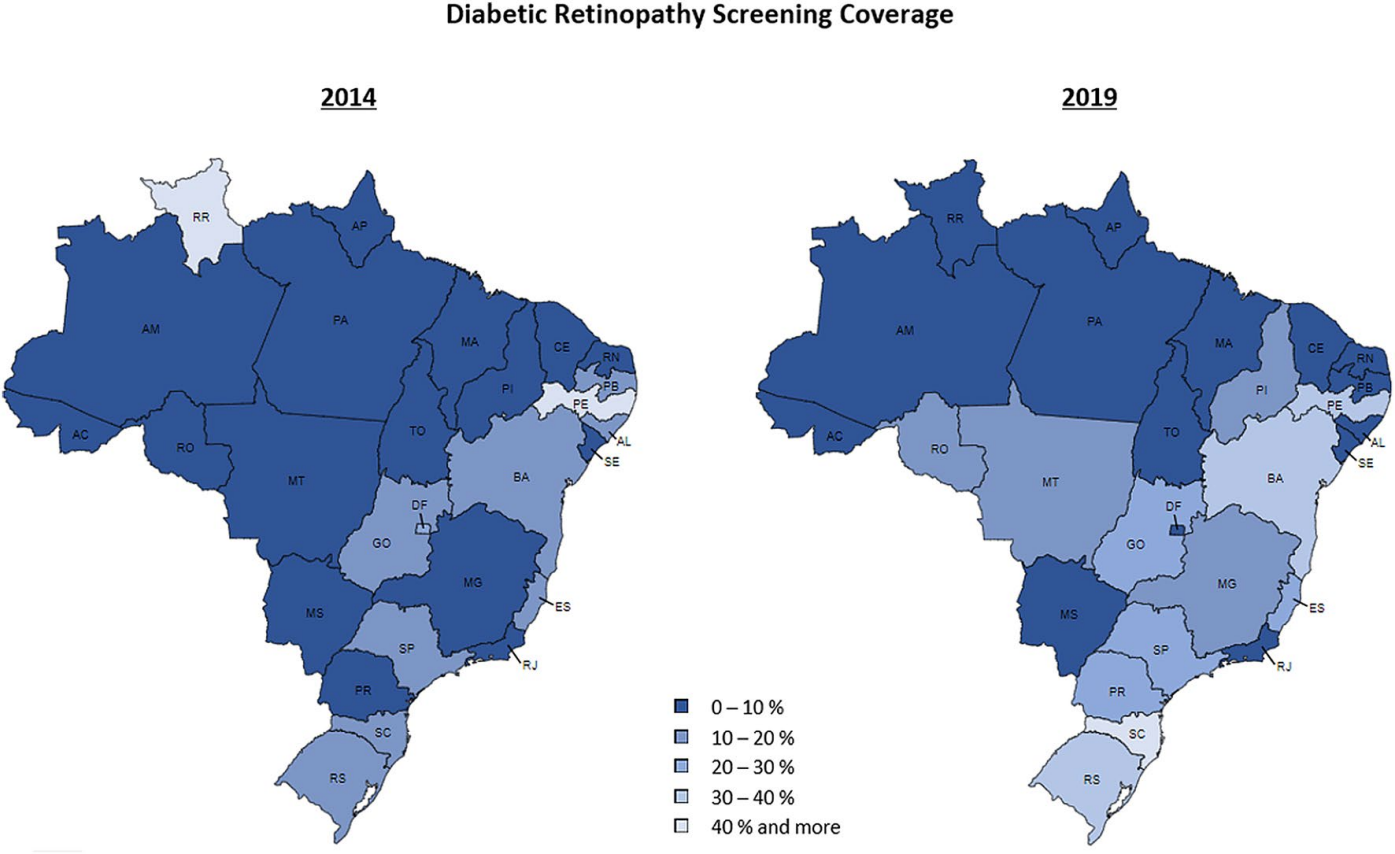
Diabetic Retinopathy Screening Coverage among Diabetic Patients through SUS in 2019, by state. Source: Go-cart.io.

South (*p* = 0.001), Southeast (*p* = 0.009), Northeast (*p* = 0.025), and Midwest (*p* = 0.004) showed increase coverage from 2014 to 2019 of 154.2%, 74.2%, 36.9%, and 36.6%, respectively. No significant changes were observed in the North region (*p* = 0.663) and it remained as the lowest coverage among all regions along the entire period. Figure 2 shows each state screening coverage in 2019. Individual data for each state along the full study period is presented on Supplementary Table S1.

Considering the total of screening procedures, clinical dilated fundus exam counted for 90.98% and color fundus photographs counted for 9.02%.

### Treatment

Along the 6 years period from 2014 to 2019, a total of 275,152 intravitreal injections, 718,342 photocoagulations, and 115,443 panretinal photocoagulations were performed through the National Health Insurance (SUS), summing 1,108,937 treatment procedures. Table 2 shows the number of treatment procedures and coverage along the years in the different regions.

**Table 2:** Number of procedures and coverage of Diabetic Retinopathy Treatment among Diabetic Patients through SUS from 2014 to 2019 according to the region.

	North	Northeast	Southeast	South	Midwest	All
2014	4,736 12.1%	22,626 17.0%	93,748 36.4%	25,625 31.0%	6,613 18.5%	153,348 27.7%
2015	5,579 15.5%	21,048 16.2%	107,210 46.9%	25,659 33.6%	7,792 20.9%	167,288 32.4%
2016	7,420 19.9%	21,744 13.7%	108,140 36.4%	24,207 28.4%	6,955 16.0%	168,496 26.9%
2017	8,877 22.8%	24,715 18.6%	116,762 46.9%	28,152 39.3%	7,542 19.6%	186,048 34.5%
2018	13,857 25.2%	26,251 18.7%	109,863 45.7%	39,892 55.0%	9,746 26.6%	199,609 36.4%
2019	16,204 42.7%	30,324 21.8%	133,341 55.7%	44,432 58.7%	9,847 26.1%	234,148 44.1%

First row: Number of diabetic retinopathy treatment procedures.

Second row: Diabetic Retinopathy Treatment Coverage.

**Figure 3:**
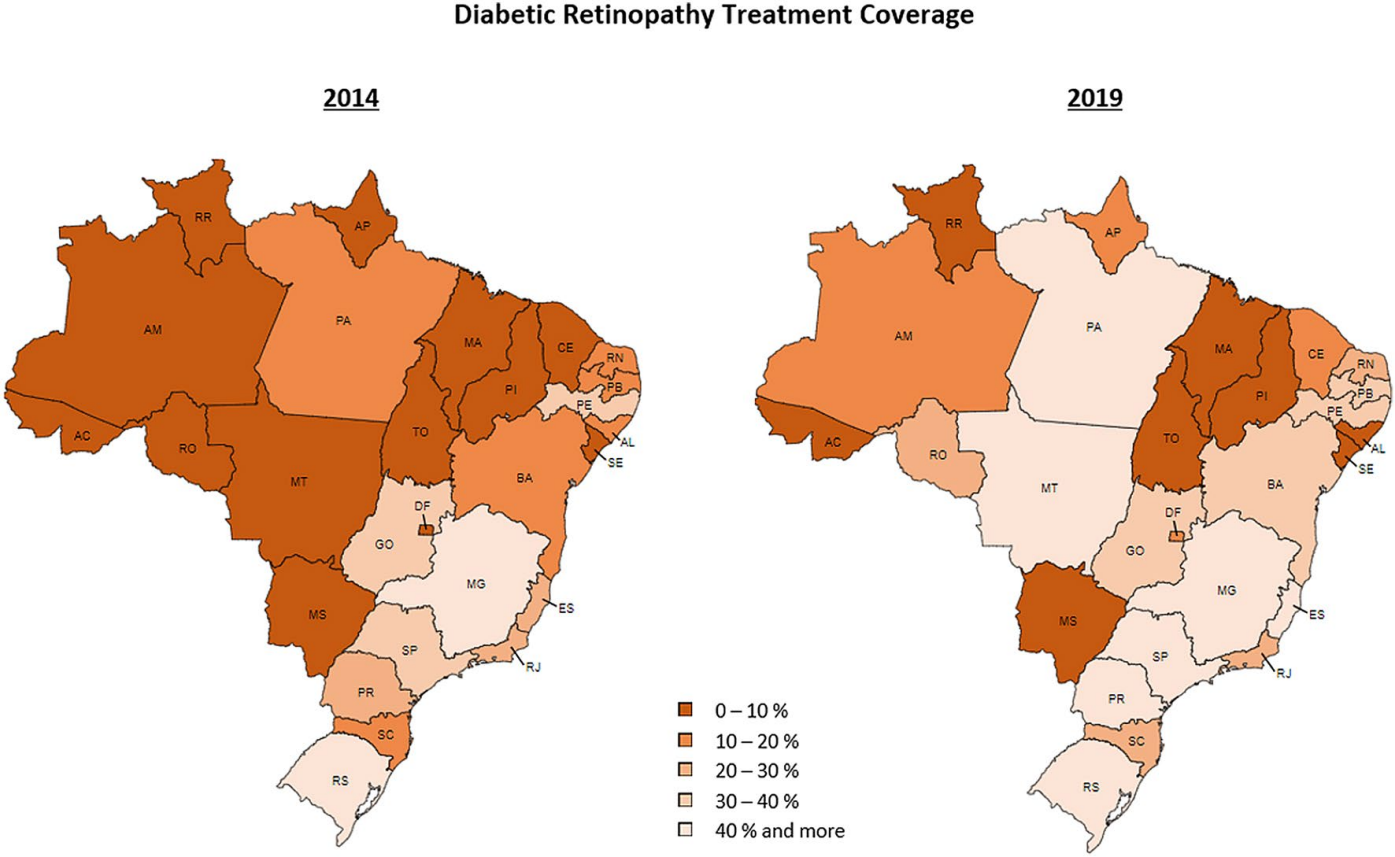
Diabetic Retinopathy Treatment Coverage among Diabetic Patients through SUS in 2014 and 2019, by state. Source: Go-cart.io.

Overall, there was a significant increase on the treatment coverage from 2014 to 2019 of 59.2% (*p* = 0.001). North (*p* < 0.001), South (*p* < 0.001), Southeast (*p* = 0.022), Midwest (p = 0.027), and Northeast (*p* = 0.043) showed increase coverages of 252.3%, 89.3%, 53.1%, 41.3%, and 28.6%, respectively. Figure 3 shows each state treatment coverage in 2019. Individual data for each state along the full study period is presented on Supplementary Table S2.

Table 3 shows each region spatial treatment coverage for each year according to the performed procedure.

**Table 3:** Spatial treatment coverage for diabetic retinopathy according to year and type of procedure.

Region	Intravitreal Injection						Photocoagulation						Panretinal photocoagulation					
	2014 (%)	2015 (%)	2016 (%)	2017 (%)	2018 (%)	2019 (%)	2014 (%)	2015 (%)	2016 (%)	2017 (%)	2018 (%)	2019 (%)	2014 (%)	2015 (%)	2016 (%)	2017 (%)	2018 (%)	2019 (%)
North	94.97	99.07	97.97	97.65	95.41	97.75	97.86	98.92	99.47	99.82	99.74	99.78	97.74	98.48	97.89	97.51	96.17	98.18
Northeast	99.81	99.93	99.92	100.00	99.97	99.89	99.97	99.81	100.02	100.03	100.01	100.03	100.00	100.14	100.21	99.90	99.83	99.84
Southeast	100.18	99.96	100.09	100.05	100.02	100.11	100.03	100.02	100.00	100.01	100.02	100.04	100.16	99.97	99.95	100.05	100.00	100.04
South	99.89	100.00	99.90	99.79	99.89	99.98	99.97	100.00	99.94	99.92	99.97	99.99	99.95	100.05	99.91	99.87	99.98	100.05
Midwest	100.43	100.61	100.15	100.52	101.12	100.06	101.52	101.12	101.10	100.59	100.60	99.83	101.82	101.46	103.41	102.92	103.19	101.56

Values below 100% indicates that not enough procedures had been offered in the region so patients needed to perform the procedure in a different region. Most of the time, Southeast and Midwest absorbed the demand for service from the North, Northeast and South. The regional discrepancies become more evident when analyzing individual state data as presented on Supplementary Table S3.

## Discussion

The prevalence of DM follows the global population life expectancy increasing, so that the International Diabetes Federation estimates a prevalence of 10.9% of the population affected by 2045, summing more than 700 million diabetic patients^1^. With the advances on diabetes overall care and awareness of lifestyle changing importance, the lifespan of people living with DM is expected to consequently increases. In that sense, a burden of micro and macrovascular complications associated with disease duration and demand for treatment are expected in the next years.

The most recent report from the Global Burden of Disease (GBD) Study indicates DR as the fifth leading cause of blindness and of moderate to severe vision impairment in adults aged 50 years and older. When evaluating the last 30 years, while significant decreases on the age-standardized global prevalence for blindness due to cataract, uncorrected refractive error, glaucoma, and age-related macular degeneration were noticed, the prevalence of blindness due to DR was the only one to significantly increase from 14.9% in 1990 to 18.5% in 2020. This is a particularly concerning scenario as, in comparison to the leading causes of visual impairment and blindness (i.e. cataract and uncorrected refractive error), the management of DR requires a greater amount of both human and technological resources, including trained ophthalmologists with experience in laser and surgery^5^.

Screening for DR has been proved to be effective on avoiding visual loss as it detects referable cases for timely full ophthalmic examination and treatment^19^. Different guidelines have been proposed along the years aiming to reach the ideal screening protocol considering both the exam procedure and the timeline design. In Brazil, a patient will only go through a DR screening procedure at the public health system by referral from a primary care doctor, an endocrinologist or an ophthalmologist. Similarly, a patient will only go through a DR treatment procedure by referral from an ophthalmologist, after a clinical evaluation.

Our study showed a lower coverage of annual DR screening in Brazil with an overall rate of 21.2% of diabetic patients being examined in 2019. No previous studies were performed in other countries with overall national data, however, previous reports with specific communities in Tanzania and in England have shown DR annual screening uptake rates of 28.8% and 82.4%, respectively^20, 21^. Factors associated with higher compliance to DR screening include older age, higher educational level, and living closer to the health facility^20–23^.

Another interest finding on the current analysis is the predominance of clinical dilated fundus exam while color fundus photographs represented only 9.0% of the screening procedures. Fundus photographs in combination with telemedicine protocols have the potential to benefit a large amount of patients improving a screening program cost-effectiveness, particularly in resource-constrained health care settings^24, 25^. Different health care workers are able to operate retinal imaging devices, not limiting the procedure to highly specialized staff^26^. Images acquired can then be shared with and graded, remotely, by ophthalmologists or through artificial intelligence algorithms. Several studies have proven the validity of such procedure in comparison to clinical dilated fundus exam and therefore this model should be implanted in order to improve the DR screening programs^27–29^.

A DR screening cost-effectiveness is also highly influenced by the frequency of the clinical examinations and/or retinal imaging^30^. Fixed annual screening programs have the challenge of the ever-increasing number of diabetic (and therefore) eligible patients requiring higher budgets each year^31–33^ and the challenge of lower patient compliance as a large proportion of screened patients who has the same result of no detected retinopathy every year tends to skip the visits on the following years^34^. Several studies in European countries have shown successful results on extending the screening interval from annual to every 2 or 3 years for patients with no evidence of retinopathy at the exam^35–37^. In that sense, differentiating patients into low-risk and high-risk groups to determine the ideal timeline of screening has the potential to further improve cost-effectiveness, mainly in low-resources settings.

Previous studies performed with individual sampling have shown DR treatment coverages among diabetic patients ranging from of 20% in Oman to 79% in Australia^38, 39^. Even though we searched DATASUS database for therapeutic procedures that are usually part of the treatment of diabetic retinopathy, the applied method carries a limitation that overestimates the real number of patients treated for diabetic retinopathy, since there are other retinal diseases treated with the same procedures; unfortunately, it is not possible to identify each patients’ diagnosis on DATASUS database. Hence, the number of procedures correspond to the whole amount of patients with a myriad of retinal diseases treated with the above mentioned procedures, including not only DR but also age-related macular degeneration and retinal vascular occlusions, retinal tears, among others. Additionally, because no patient is identified, and since the same patient may have undergone more than one therapeutic procedure, the rate of coverage is further overestimated. Consequently, the poor coverage rate found in the present study is probably even lower, which further aggravates the DR treatment coverage landscape in Brazil’s public health system.

Our search did not include vitrectomy as a therapeutic procedure for DR patients because this kind of surgery is performed in only a few services throughout Brazil’s public health system, and it is not mainly performed for DR treatment, but rather for rhegmatogenous retinal detachment. Additionally, currently there is no reliable data on the rate of procedures performed to address the treatment of DR. Elsewhere, only a small fraction of such procedures are performed for the treatment of DR in underserved countries^40^; finally, most diabetic vitrectomies are performed along with laser pan photocoagulation, which has already been considered in our search.

Significant disparities according to the region were observed. Brazil is a country with continental extension with substantial socioeconomic regional disparities that also influences the ophthalmologist’s distribution throughout the country. According to the World Health Organization (WHO), the ideal scenario of ocular health care within a population is a rate of at least 1 ophthalmologist per 17,000 habitants. The most recent census promoted by the Brazilian Council of Ophthalmology in 2019 shows that the country counts with 20,455 ophthalmologists which results in 1 professional per 9,224 population, in accordance to the WHO recommendation. When analyzing each region, however, the rates range from 1 per 7599 in the Southeast to 1 per 12,084 in the North region^41^. The disparities on concentration of ophthalmologists in the country may impact both the frequency of procedures performed and the spatial coverage in each region. A recent study on risk of developing DM in the next 10 years in the Brazilian population indicated a significantly higher risk in the North region so that the current observed disparities are expected to increase if no intervention is designed^42^.

In conclusion, despite an improvement along the past 6 years, both screening and treatment for diabetic retinopathy in diabetes patients are ineffective in Brazil, with substantial differences among the regions. Screening programs should focus on patients awareness, flexible timeline planning according to risk, and use of telemedicine protocols including fundus photograph and artificial intelligence. Moreover, public health policies should address the unequal distribution of human and technological resources throughout the country in order to offer the same health care conditions of screening and treatment to patients regardless their geographical location.

## Supplementary Information

Supplementary Information.
