# Supplementary material for: Diabetic retinopathy screening and treatment through the Brazilian National Health Insurance
Source: Sci Rep. 2022 Aug 17;12:13941. doi: 10.1038/s41598-022-18054-6 (PMC9385734; doi:10.1038/s41598-022-18054-6)
Supplement: Supplementary file 1 — Supplementary Information. [file 41598_2022_18054_MOESM1_ESM.docx]

**SUPPLEMENTAR MATERIAL**

Table S1. Diabetic Retinopathy Screening Coverage among Diabetic Patients through SUS from 2014 to 2019 according to the region

| **Region** | **State** | **2014** | **2015** | **2016** | **2017** | **2018** | **2019** |
| --- | --- | --- | --- | --- | --- | --- | --- |
| **N** | Rondônia | 5.0% | 4.4% | 2.5% | 2.8% | 4.0% | 15.7% |
| **N** | Acre | 1.8% | 3.0% | 2.2% | 1.5% | 0.0% | 0.4% |
| **N** | Amazonas | 9.7% | 9.3% | 6.6% | 7.3% | 6.5% | 7.6% |
| **N** | Roraima | 71.3% | 74.8% | 39.7% | 22.4% | 31.7% | 5.9% |
| **N** | Pará | 5.9% | 7.7% | 8.2% | 9.2% | 8.7% | 9.7% |
| **N** | Amapá | 0.3% | 1.0% | 1.6% | 1.4% | 2.7% | 3.0% |
| **N** | Tocantins | 6.5% | 13.1% | 11.7% | 10.8% | 7.0% | 8.5% |
|  | **Overall N** | **7.8%** | **9.1%** | **7.8%** | **7.9%** | **5.7%** | **8.8%** |
| **NE** | Maranhão | 4.6% | 4.3% | 4.4% | 7.8% | 6.8% | 8.1% |
| **NE** | Piauí | 9.8% | 10.3% | 10.3% | 10.1% | 11.9% | 19.7% |
| **NE** | Ceará | 3.8% | 5.4% | 4.2% | 3.9% | 3.9% | 5.2% |
| **NE** | Rio Grande do Norte | 4.2% | 3.5% | 2.7% | 3.6% | 4.1% | 3.3% |
| **NE** | Paraíba | 13.0% | 16.8% | 14.1% | 11.2% | 7.4% | 8.4% |
| **NE** | Pernambuco | 40.4% | 48.0% | 43.9% | 55.9% | 55.0% | 47.2% |
| **NE** | Alagoas | 14.2% | 13.9% | 15.1% | 12.5% | 3.0% | 3.4% |
| **NE** | Sergipe | 6.4% | 8.6% | 6.4% | 7.5% | 8.9% | 8.0% |
| **NE** | Bahia | 13.8% | 12.0% | 12.2% | 16.5% | 21.5% | 30.1% |
|  | **Overall NE** | **14.9%** | **16.7%** | **15.5%** | **18.3%** | **17.4%** | **20.4%** |
| **SE** | Minas Gerais | 7.5% | 10.4% | 7.8% | 10.0% | 12.1% | 13.8% |
| **SE** | Espírito Santo | 16.6% | 18.1% | 14.7% | 17.3% | 24.8% | 27.7% |
| **SE** | Rio de Janeiro | 5.4% | 7.5% | 7.0% | 7.7% | 7.1% | 7.4% |
| **SE** | São Paulo | 15.6% | 21.6% | 18.4% | 24.5% | 27.0% | 29.0% |
|  | **Overall SE** | **12.0%** | **15.8%** | **13.4%** | **17.3%** | **18.8%** | **20.9%** |
| **S** | Paraná | 9.4% | 13.9% | 10.8% | 17.3% | 19.5% | 20.5% |
| **S** | Santa Catarina | 12.8% | 23.4% | 27.8% | 35.4% | 35.2% | 52.2% |
| **S** | Rio Grande do Sul | 16.8% | 23.8% | 26.4% | 30.0% | 32.9% | 35.3% |
|  | **Overall S** | **13.1%** | **20.2%** | **19.9%** | **26.0%** | **28.6%** | **33.3%** |
| **MW** | Mato Grosso do Sul | 3.9% | 16.8% | 21.1% | 7.2% | 13.8% | 16.4% |
| **MW** | Mato Grosso | 1.5% | 2.1% | 1.7% | 9.1% | 4.2% | 1.5% |
| **MW** | Goiás | 14.9% | 16.8% | 20.2% | 23.3% | 29.0% | 28.0% |
| **MW** | Distrito Federal | 23.5% | 1.9% | 1.5% | 2.3% | 3.9% | 3.4% |
|  | **Overall MW** | **11.2%** | **11.2%** | **12.8%** | **13.6%** | **15.9%** | **15.3%** |
|  | **Overall Brazil** | **12.4%** | **15.6%** | **14.4%** | **17.5%** | **18.1%** | **21.2%** |

*N = North; NE = Northeast; SE = Southeast; S = South; MW = Midwest

Table S2. Diabetic Retinopathy Treatment Coverage among Diabetic Patients through SUS from 2014 to 2019 according to the region

| **Region** | **State** | **2014** | **2015** | **2016** | **2017** | **2018** | **2019** |
| --- | --- | --- | --- | --- | --- | --- | --- |
| **N** | Rondônia | 4.3% | 5.4% | 12.2% | 8.3% | 14.3% | 21.4% |
| **N** | Acre | 0.2% | 0.2% | 0.4% | 0.2% | 0.0% | 0.8% |
| **N** | Amazonas | 2.1% | 6.4% | 9.8% | 10.1% | 7.9% | 19.5% |
| **N** | Roraima | 64.7% | 70.3% | 9.7% | 0.1% | 1.7% | 6.3% |
| **N** | Pará | 18.3% | 23.6% | 32.2% | 41.8% | 60.4% | 67.8% |
| **N** | Amapá | 1.3% | 0.7% | 0.1% | 3.6% | 3.9% | 11.9% |
| **N** | Tocantins | 4.1% | 5.5% | 3.9% | 4.9% | 8.9% | 8.6% |
|  | **Overall N** | **12.1%** | **15.5%** | **19.9%** | **22.8%** | **25.2%** | **42.7%** |
| **NE** | Maranhão | 7.9% | 3.7% | 2.4% | 3.7% | 5.8% | 7.7% |
| **NE** | Piauí | 1.9% | 2.0% | 2.7% | 4.3% | 4.6% | 4.7% |
| **NE** | Ceará | 5.8% | 9.2% | 5.4% | 7.2% | 8.7% | 11.1% |
| **NE** | Rio Grande do Norte | 17.8% | 20.0% | 18.2% | 30.9% | 25.8% | 21.0% |
| **NE** | Paraíba | 17.0% | 18.2% | 17.3% | 13.0% | 20.9% | 33.8% |
| **NE** | Pernambuco | 38.8% | 36.3% | 24.9% | 36.5% | 30.2% | 31.2% |
| **NE** | Alagoas | 13.0% | 13.3% | 13.6% | 14.3% | 13.0% | 9.2% |
| **NE** | Sergipe | 1.4% | 1.0% | 2.2% | 0.0% | 0.2% | 2.4% |
| **NE** | Bahia | 17.8% | 14.3% | 16.5% | 24.8% | 30.6% | 35.4% |
|  | **Overall NE** | **17.0%** | **16.2%** | **13.7%** | **18.6%** | **18.7%** | **21.8%** |
| **SE** | Minas Gerais | 41.6% | 48.0% | 32.0% | 42.7% | 38.3% | 46.7% |
| **SE** | Espírito Santo | 28.1% | 36.5% | 14.8% | 31.0% | 65.4% | 81.5% |
| **SE** | Rio de Janeiro | 27.9% | 25.4% | 18.2% | 23.7% | 14.5% | 24.3% |
| **SE** | São Paulo | 38.2% | 56.3% | 47.3% | 59.5% | 62.4% | 70.5% |
|  | **Overall SE** | **36.4%** | **46.9%** | **36.4%** | **46.9%** | **45.7%** | **55.7%** |
| **S** | Paraná | 25.0% | 31.4% | 24.0% | 35.5% | 67.8% | 77.7% |
| **S** | Santa Catarina | 16.5% | 27.5% | 26.0% | 31.1% | 31.3% | 29.3% |
| **S** | Rio Grande do Sul | 45.1% | 38.2% | 34.4% | 46.2% | 56.1% | 56.4% |
|  | **Overall S** | **31.0%** | **33.6%** | **28.4%** | **39.3%** | **55.0%** | **58.7%** |
| **MW** | Mato Grosso do Sul | 8.4% | 19.0% | 17.9% | 22.0% | 29.3% | 40.6% |
| **MW** | Mato Grosso | 0.0% | 0.7% | 0.1% | 0.3% | 0.2% | 0.9% |
| **MW** | Goiás | 38.8% | 32.9% | 25.1% | 28.6% | 39.8% | 38.3% |
| **MW** | Distrito Federal | 9.0% | 15.8% | 12.1% | 14.7% | 26.8% | 17.8% |
|  | **Overal MW** | **18.5%** | **20.9%** | **16.0%** | **19.6%** | **26.6%** | **26.1%** |
|  | **Overall Brazil** | **27.7%** | **32.4%** | **26.9%** | **34.5%** | **36.4%** | **44.1%** |

*N = North; NE = Northeast; SE = Southeast; S = South; MW = Midwest

Table S3. Spatial treatment coverage for diabetic retinopathy for each state according to year and type of procedure

| **State** | **Intravitreal Injection** | | | | | | **Photocoagulation** | | | | | | **Panretinal photocoagulation** | | | | | |
| --- | --- | --- | --- | --- | --- | --- | --- | --- | --- | --- | --- | --- | --- | --- | --- | --- | --- | --- |
|  | 2014 | 2015 | 2016 | 2017 | 2018 | 2019 | 2014 | 2015 | 2016 | 2017 | 2018 | 2019 | 2014 | 2015 | 2016 | 2017 | 2018 | 2019 |
| Rondônia | 98.43% | 101.70% | 101.60% | 99.48% | 100.37% | 100.67% | 0.00% | 0.00% | 0.00% | 0.00% | 0.00% | 66.67% | 100.00% | 0.00% | 0.00% | 0.00% | 0.00% | 0.00% |
| Acre | 0.00% | 0.00% | 0.00% | 0.00% | 0.00% | 0.00% | 0.00% | 0.00% | 0.00% | 0.00% | 0.00% | 0.00% | 0.00% | 0.00% | 0.00% | 0.00% | 0.00% | 0.00% |
| Amazonas | 0.00% | 99.56% | 33.33% | 66.67% | 92.16% | 97.33% | 98.21% | 98.37% | 100.00% | 100.00% | 100.00% | 99.70% | 100.00% | 100.00% | 100.00% | 100.00% | 100.00% | 100.00% |
| Roraima | 0.00% | 0.00% | 0.00% | 0.00% | 100.00% | 0.00% | 0.00% | 0.00% | 0.00% | 0.00% | 0.00% | 0.00% | 99.81% | 100.00% | 98.75% | 0.00% | 100.00% | 100.00% |
| Pará | 96.86% | 100.00% | 99.53% | 99.36% | 98.43% | 97.98% | 100.04% | 99.93% | 99.98% | 100.62% | 100.40% | 100.76% | 100.43% | 99.91% | 100.00% | 101.51% | 100.46% | 101.17% |
| Amapá | 0.00% | 0.00% | 0.00% | 0.00% | 77.78% | 95.45% | 0.00% | 0.00% | 0.00% | 0.00% | 0.00% | 0.00% | 0.00% | 0.00% | 0.00% | 0.00% | 0.00% | 0.00% |
| Tocantins | 100.00% | 0.00% | 0.00% | 33.33% | 20.59% | 50.00% | 15.00% | 63.24% | 80.82% | 79.59% | 85.71% | 88.14% | 0.00% | 39.02% | 24.44% | 22.95% | 1.92% | 17.65% |
| Maranhão | 25.00% | 87.18% | 92.06% | 91.04% | 93.18% | 95.24% | 99.86% | 92.31% | 89.86% | 96.92% | 98.26% | 97.08% | 98.04% | 99.32% | 100.00% | 100.00% | 100.00% | 99.78% |
| Piauí | 70.00% | 100.00% | 75.00% | 88.24% | 79.17% | 84.21% | 101.10% | 98.10% | 96.61% | 99.18% | 100.68% | 97.62% | 100.00% | 76.92% | 94.12% | 100.00% | 100.00% | 100.00% |
| Ceará | 100.48% | 98.46% | 98.95% | 100.88% | 100.59% | 100.47% | 99.78% | 100.40% | 100.62% | 99.74% | 99.85% | 100.49% | 0.00% | 0.00% | 0.00% | 0.00% | 0.00% | 95.12% |
| R. Gde do Norte | 98.09% | 99.65% | 100.16% | 99.95% | 99.95% | 99.93% | 96.20% | 98.30% | 99.56% | 99.62% | 98.59% | 99.46% | 100.00% | 100.12% | 100.00% | 100.00% | 100.00% | 100.00% |
| Paraíba | 92.55% | 94.98% | 91.77% | 92.91% | 97.16% | 96.66% | 99.88% | 92.02% | 90.41% | 62.20% | 96.06% | 97.68% | 21.74% | 90.35% | 95.59% | 99.24% | 99.60% | 99.52% |
| Pernambuco | 102.38% | 102.23% | 102.60% | 101.77% | 102.65% | 101.43% | 100.84% | 101.18% | 102.13% | 101.86% | 101.13% | 101.32% | 103.26% | 102.34% | 104.04% | 100.75% | 101.71% | 100.93% |
| Alagoas | 97.20% | 99.05% | 100.00% | 99.04% | 94.67% | 97.70% | 99.72% | 100.00% | 99.26% | 99.62% | 99.46% | 98.19% | 75.56% | 82.86% | 96.77% | 100.00% | 100.00% | 98.46% |
| Sergipe | 0.00% | 0.00% | 0.00% | 0.00% | 0.00% | 0.00% | 100.00% | 102.70% | 100.00% | 0.00% | 50.00% | 100.00% | 100.00% | 100.00% | 100.00% | 0.00% | 40.00% | 100.00% |
| Bahia | 98.09% | 98.76% | 98.06% | 99.25% | 98.84% | 99.46% | 99.59% | 99.63% | 99.87% | 99.88% | 99.89% | 100.01% | 81.82% | 94.37% | 99.33% | 97.53% | 96.83% | 98.88% |
| Minas Gerais | 96.65% | 98.79% | 98.17% | 97.91% | 97.75% | 97.81% | 99.60% | 99.55% | 99.48% | 99.15% | 99.41% | 99.34% | 97.73% | 98.14% | 97.35% | 98.24% | 98.37% | 98.31% |
| Espírito Santo | 100.00% | 85.42% | 82.98% | 100.19% | 100.17% | 100.06% | 100.81% | 101.01% | 99.78% | 100.30% | 99.95% | 100.03% | 101.67% | 99.89% | 100.00% | 100.40% | 100.59% | 100.00% |
| Rio de Janeiro | 95.35% | 99.06% | 86.71% | 95.26% | 98.24% | 99.91% | 99.95% | 100.01% | 99.85% | 99.88% | 99.86% | 100.00% | 100.83% | 100.23% | 100.30% | 100.17% | 99.78% | 100.04% |
| São Paulo | 101.04% | 100.43% | 100.64% | 100.78% | 100.69% | 100.83% | 100.20% | 100.17% | 100.22% | 100.33% | 100.22% | 100.28% | 100.14% | 100.71% | 101.05% | 100.58% | 100.26% | 100.34% |
| Paraná | 99.14% | 100.00% | 99.84% | 100.27% | 100.04% | 99.94% | 100.07% | 100.26% | 100.03% | 99.87% | 100.11% | 99.98% | 99.94% | 100.16% | 99.85% | 100.36% | 100.08% | 100.04% |
| Santa Catarina | 99.93% | 100.00% | 100.00% | 99.57% | 99.92% | 99.83% | 99.44% | 99.16% | 99.35% | 99.69% | 98.76% | 99.50% | 100.00% | 100.00% | 99.58% | 97.84% | 99.79% | 100.10% |
| R. Gde do Sul | 100.00% | 100.00% | 99.82% | 99.57% | 99.73% | 100.16% | 99.97% | 100.01% | 100.00% | 99.99% | 99.98% | 100.03% | 99.94% | 99.94% | 100.00% | 100.00% | 99.95% | 100.04% |
| Mt Grosso Sul | 99.55% | 100.82% | 99.15% | 99.86% | 99.61% | 100.00% | 97.74% | 96.97% | 100.00% | 99.50% | 99.64% | 98.98% | 0.00% | 100.00% | 100.00% | 100.00% | 100.00% | 100.00% |
| Mt Grosso | 100.00% | 97.96% | 100.00% | 100.00% | 0.00% | 62.96% | 0.00% | 0.00% | 0.00% | 0.00% | 0.00% | 58.82% | 0.00% | 0.00% | 100.00% | 0.00% | 33.33% | 0.00% |
| Goiás | 96.23% | 94.74% | 94.42% | 94.49% | 96.38% | 91.50% | 101.12% | 101.41% | 100.86% | 99.56% | 97.86% | 98.41% | 102.13% | 100.31% | 101.27% | 101.27% | 99.79% | 99.52% |
| Distrito Federal | 108.22% | 109.60% | 109.88% | 113.87% | 110.87% | 114.40% | 125.81% | 117.86% | 115.53% | 114.86% | 118.42% | 125.08% | 0.00% | 117.80% | 122.03% | 118.33% | 126.38% | 115.95% |
